# In vivo genome-wide profiling reveals a tissue-specific role for 5-formylcytosine

**DOI:** 10.1186/s13059-016-1001-5

**Published:** 2016-06-29

**Authors:** Mario Iurlaro, Gordon R. McInroy, Heather E. Burgess, Wendy Dean, Eun-Ang Raiber, Martin Bachman, Dario Beraldi, Shankar Balasubramanian, Wolf Reik

**Affiliations:** The Babraham Institute, Epigenetics Programme, Cambridge, CB22 3AT UK; Department of Chemistry, University of Cambridge, Lensfield Road, Cambridge, CB2 1EW UK; Cancer Research UK, Cambridge Institute, Li Ka Shing Centre, Cambridge, CB2 0RE UK; Present Address: Discovery Sciences, AstraZeneca, Alderley Park, Macclesfield, SK10 4TG UK; School of Clinical Medicine, University of Cambridge, Cambridge, CB2 0SP UK; Centre for Trophoblast Research, University of Cambridge, Cambridge, CB2 3EG UK; Wellcome Trust Sanger Institute, Hinxton, CB10 1SA UK

## Abstract

**Background:**

Genome-wide methylation of cytosine can be modulated in the presence of TET and thymine DNA glycosylase (TDG) enzymes. TET is able to oxidise 5-methylcytosine (5mC) to 5-hydroxymethylcytosine (5hmC), 5-formylcytosine (5fC) and 5-carboxylcytosine (5caC). TDG can excise the oxidative products 5fC and 5caC, initiating base excision repair. These modified bases are stable and detectable in the genome, suggesting that they could have epigenetic functions in their own right. However, functional investigation of the genome-wide distribution of 5fC has been restricted to cell culture-based systems, while its in vivo profile remains unknown.

**Results:**

Here, we describe the first analysis of the in vivo genome-wide profile of 5fC across a range of tissues from both wild-type and *Tdg*-deficient E11.5 mouse embryos. Changes in the formylation profile of cytosine upon depletion of TDG suggest TET/TDG-mediated active demethylation occurs preferentially at intron-exon boundaries and reveals a major role for TDG in shaping 5fC distribution at CpG islands. Moreover, we find that active enhancer regions specifically exhibit high levels of 5fC, resulting in characteristic tissue-diagnostic patterns, which suggest a role in embryonic development.

**Conclusions:**

The tissue-specific distribution of 5fC can be regulated by the collective contribution of TET-mediated oxidation and excision by TDG. The in vivo profile of 5fC during embryonic development resembles that of embryonic stem cells, sharing key features including enrichment of 5fC in enhancer and intragenic regions. Additionally, by investigating mouse embryo 5fC profiles in a tissue-specific manner, we identify targeted enrichment at active enhancers involved in tissue development.

**Electronic supplementary material:**

The online version of this article (doi:10.1186/s13059-016-1001-5) contains supplementary material, which is available to authorized users.

## Background

Active DNA demethylation in mammals can be achieved via iterative oxidation of 5-methylcytosine (5mC) by one or more of the TET enzymes to generate 5-hydroxymethylcytosine (5hmC), 5-formylcytosine (5fC) and 5-carboxylcytosine (5caC) [1–5]. 5fC and 5caC are recognised and excised by the thymine DNA glycosylase (TDG) enzyme, initiating base excision repair (BER). Alternatively, 5fC may be lost passively during DNA replication [5, 6]. Following the initial identification of 5fC as an oxidative product of 5mC, 5fC has been detected in a variety of tissues and cell systems, albeit at relatively low levels compared to 5mC and 5hmC [4, 7]. Recently we demonstrated that 5fC is not only detectable but also stable in vivo [8], lending credence to a biological role for 5fC beyond that of a demethylation intermediate. Indeed, several proteins have been found to specifically bind 5fC in mouse embryonic stem cells (mESCs) and neural precursor cells (NPCs) with putative roles in transcription, chromatin regulation and DNA repair [9, 10]. The mechanisms by which 5fC affects these processes are not known, but it has recently been proposed that changes in the physical properties of the DNA double-stranded helix may be involved [11].

In the past three years, a number of different methods have been described for the mapping of 5fC in the genome of mESCs. These methods fall broadly into two categories: (1) affinity enrichment methods relying on antibody or chemical pull-downs [12–14]; and (2) base-resolution methods based on modifications to the classical bisulphite sequencing (BS-seq) technique [13, 15–17]. Recently two bisulphite-free base-resolution methods have been described that employ either the change in base-pairing properties (C to T) upon chemical modification [18] or the use of a specific restriction enzyme followed by an enrichment step [19]. Despite the methodological differences between these techniques, a consistent picture of the distribution of 5fC in mESCs is emerging. 5fC is enriched at enhancers, repetitive elements and exons, but how this relates to its distribution in tissues of a whole organism is currently unexplored. Considering the dynamic nature of the modification and its enrichment at regulatory regions, this becomes extremely relevant especially in the context of development, where epigenetic modifications are subject to changes.

Here we present the first in vivo genome-wide profile of 5fC, in tissues of the early mouse embryo. In particular, we describe the distribution of 5fC in the hindbrain, heart, liver and carcass of embryonic day (E) 11.5 wild-type (WT) and *Tdg* null embryos. The use of *Tdg* null embryos enables the identification of sites marked by active demethylation driven by TET/TDG activity in these tissues, highlighting a potential role for 5fC in tissue development and differentiation.

## Results and discussion

### Genomic levels of 5fC are heterogeneous in mid-gestation staged embryos

**Fig. 1:**
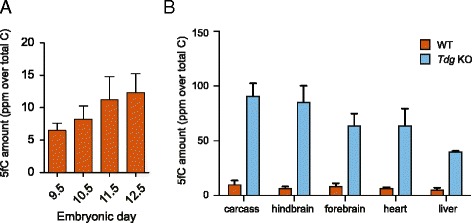
Genomic 5fC is heterogeneous in early embryos. **a** LC/MS quantification of genomic 5fC levels in mid-gestation embryos. Displayed is the average of at least three biological replicates with standard deviation. Results are expressed as ppm (part per million over total of cytosines). **b** LC/MS quantification of genomic 5fC levels in dissected tissues from WT (*brown*) and *Tdg* knockout (*light blue*) E11.5 embryos. Displayed is the average of three biological replicates with standard deviation. Results are expressed as ppm (part per million over total of cytosines)

We quantified genomic levels of 5fC from DNA extracted from whole embryos at various developmental stages between E9.5 and E12.5 by liquid chromatography–mass spectrometry (LC/MS) (Fig. 1a). We found that in mid-gestational embryos, 5fC is present at levels comparable to mouse ES cells, reaching a maximum at E11.5–E12.5. Mice lacking the TDG enzyme show substantially higher levels of 5fC (approximately a sevenfold increase) in accordance with its ability to excise 5fC (Additional file 1: Figure S1A) [6]. As previously reported [20, 21], *Tdg* null mice displayed embryonic lethality after E11.5 with a distinct phenotype characterised by haemorrhaging. We therefore decided to restrict our focus to E11.5 embryos for further analysis. We previously quantified genomic 5fC levels in dissected tissues from WT E11.5 mouse embryos by LC/MS [8]. Quantification of 5fC levels in the corresponding tissues dissected from *Tdg* null (TDG null) embryos (Fig. 1b and Additional file 1: Figure S1B) showed that levels of 5fC in the absence of *Tdg* are heterogeneous. Thus, the highest levels were detected in the carcass and in the hindbrain, where 5fC was approximately twofold higher than the concentration found in the liver, the tissue with the lowest 5fC levels in both WT and *Tdg* null embryos. Notably, this heterogeneity shows no clear correlation with the expression levels of TET and TDG enzymes measured by qPCR in WT tissues (Additional file 1: Figure S1C).

### Genome-wide profiling of 5fC with a traceless chemical probe

In order to better understand the biological role of 5fC during embryonic development we generated genome-wide profiles for 5fC in dissected tissues from E11.5 WT and *Tdg* null embryos. To this end, we developed an optimised protocol based on a previously reported 5fC-pull-down method [12], additionally employing a cleavable aldehyde-reactive biotinylation probe as previously described [22] (Additional file 1: Figure S2A). The hydroxylamine moiety exploits the unique reactivity of 5fC among the other DNA bases to install a biotin tag, enabling subsequent enrichment with streptavidin coated magnetic beads. The presence of a chemical cleavage site (Additional file 1: Figure S2B) enables removal of the biotin tag following enrichment, which alleviates PCR biases introduced by polymerase stalling at modified sites [22]. This new probe and methodology resulted in substantially increased enrichment efficiencies over 30-fold higher than the previously reported method. Through simultaneously obtaining an enhanced signal/noise ratio and increasing resolution, we were able to perform sensitive and robust profiling of 5fC from relatively small amounts of embryonic-tissue-derived genomic DNA (Additional file 1: Figure S2C). We used this capture method to systematically perform genome-wide profiling of 5fC in the hindbrain, heart, liver and carcass of E11.5 embryos. At least two biological replicates were performed for each tissue and WT and *Tdg* null samples were paired from the same litter to account for litter-induced variability. A full list of replicates is available in Additional file 1: Figure S3A. In accordance with the data obtained by LC/MS, *Tdg* null embryos show substantially higher levels of 5fC in all tissues analysed, reflected in the higher signal and increased number of 5fC peaks in all *Tdg* null samples compared to their WT counterparts. Biological replicates for the same tissues from different embryos showed an extremely high degree of similarity, suggesting a tight regulation of TET-driven formation and TDG-mediated excision of 5fC during development. Unbiased hierarchical clustering of all individual replicates accurately separates all samples derived from WT and *Tdg* null embryos, while simultaneously pairing replicates from the same tissues (Additional file 1: Figure S3B). One replicate for the *Tdg* null liver was of poor quality and therefore was excluded from the following analyses.

**Fig. 2:**
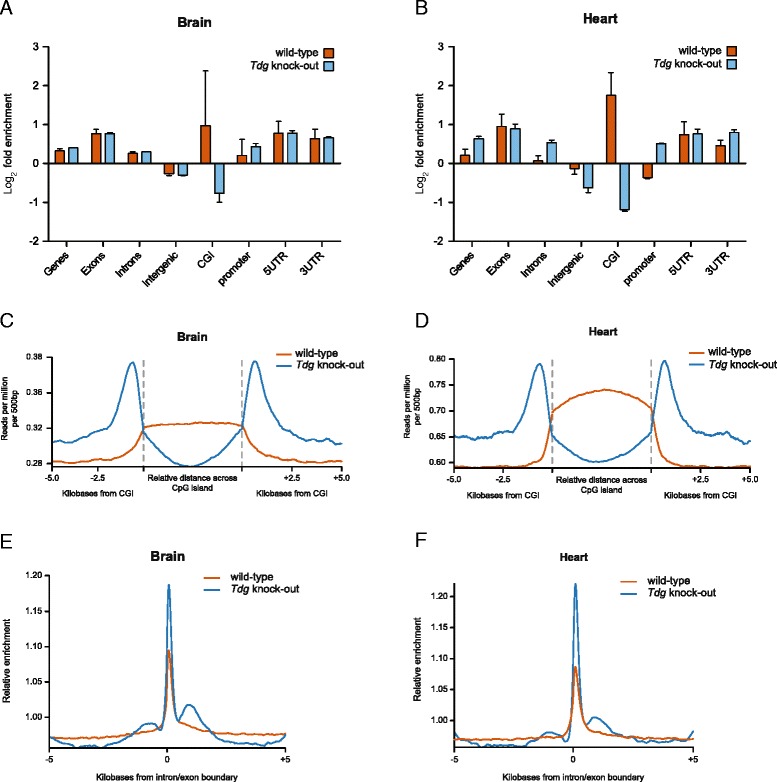
Genome-wide profiling of 5fC in vivo. **a**, **b** Enrichment of 5fC peaks over functional genomic features in E11.5 hindbrain and heart, respectively. Log_2_ fold enrichment was calculated for each replicate individually using 10,000 randomisations in a simulation procedure implemented with the GAT [21]. Plotted is the average enrichment with standard deviation. **c**, **d** Trend plot showing 5fC profile over CpG islands (+/− 5 kb) in the hindbrain and heart, respectively. **e**, **f** Trend plot showing 5fC profile over exon/intron boundaries (+/− 5 kb) in the hindbrain and heart, respectively

We then investigated how 5fC was distributed among functional features within the genome. To this end, 5fC peaks were initially called independently in each pull-down and enrichment of peaks over genomic features was calculated using the Genomic Association Tester (GAT) [23]. 5fC peaks were found to be most enriched in exons, 5′ UTRs and 3′ UTRs, while showing lesser enrichment in introns and gene bodies. Moreover, 5fC was depleted in the intergenic regions of all tissues analysed (Fig. 2a, b and Additional file 1: Figure S4). Although higher levels of 5fC in *Tdg* KO tissues result in a higher number of 5fC peaks called, the distribution of 5fC peaks over most genomic features was remarkably similar between WT and knockout samples, suggesting that in these regions TDG controls the level of 5fC without changing its pattern (Fig. 2a, b and Additional file 1: Figure S4). The only exception to this pattern was found in CpG islands (CGIs), where 5fC peaks were enriched in all WT tissues (with the exception of the carcass), but depleted in the same tissues from embryos lacking *Tdg*. It is therefore possible that TDG may be required for TET-mediated oxidation at CpG islands, as suggested by the physical interaction between TET1 and TDG [24], or that excision of 5fC by TDG is less efficient at these sites. Looking at the distribution across CGIs in more detail, we observed that in *Tdg* null embryos 5fC is absent from CGIs but enriched at flanking regions, often referred to as ‘CGI shores’ (Fig. 2c, d and Additional file 1: Figure S5). This clearly indicates a distinctive role for TDG in shaping 5fC distribution over CpG islands that is conserved across all embryonic tissues. This finding is particularly relevant since aberrant methylation of CGIs has been found in *Tdg* null mice and was suggested as a possible cause of the lethal phenotype [20, 21]. Misregulation of 5fC may be fundamentally involved in the aberrant methylation described in *Tdg* null embryos, which was previously undetectable owing to the limitation of the methylation analysis technique employed—bisulphite sequencing—to distinguish 5fC from cytosine [20, 21].

Finally, we observed a substantial enrichment for 5fC over exon/intron boundaries in both WT and knockout tissues (Fig. 2e, f and Additional file 1: Figure S6). This has also been observed for 5hmC and 5fC in embryonic stem cells and suggests a role for oxidised cytosine derivatives in regulating splicing [14, 25]. The presence of 5fC at these sites is particularly interesting, as 5fC has been found to reduce the rate and the fidelity of transcription by RNA polymerase II [26].

### 5fC is enriched at active enhancers in the mouse embryo

**Fig. 3:**
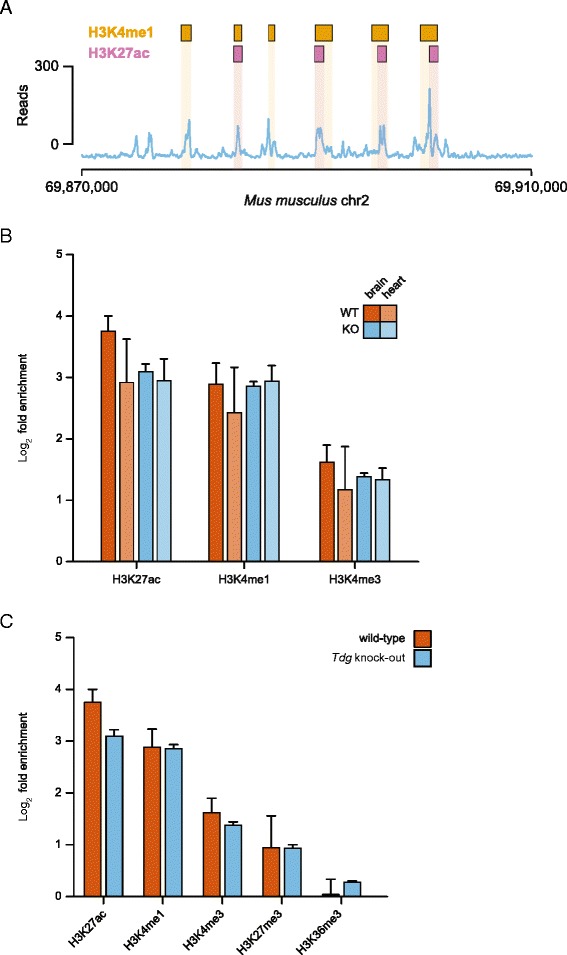
5fC is enriched at active enhancers in vivo. **a** Screenshot of a genomic region on chromosome 2 exemplifying the striking overlap of the 5fC signal with regions marked by H3K4me1 (*orange boxes*) and H3K27ac (*purple boxes*), as calculated in [27]. **b** Fold enrichment of 5fC peaks in hindbrain and heart from WT and *Tdg* null embryos over regions marked by H3K4me1, H3K4me3 and H3K27ac in the brain and heart of E14.5 embryos, respectively. Log_2_ fold enrichment was calculated for each replicate individually using 10,000 randomisations in a simulation procedure implemented with the GAT. Plotted is the average enrichment with standard deviation. **c** Comparison of 5fC peak enrichment in the hindbrain in regions marked by different histone modifications in the E14.5 mouse brain

It has been reported that the genomic distribution of 5fC in mESCs shows a significant enrichment at distal regulatory regions, mostly enhancers with high levels of mono-methylation of lysine 4 on histone H3 (H3K4me1) but low or absent acetylation of lysine 27 (H3K27ac) [13]. As they are marked by the active H3K4me1, but are still in a repressed deacetylated state, these enhancers are often referred to as ‘poised’. In order to investigate the distribution of 5fC in relation to functional enhancers in vivo, we analysed the 5fC distribution in E11.5 tissues in relation to available datasets for known histone marks generated in mouse embryos by the ENCODE consortium [27] (Additional file 2: Table S1). We found that in all tissues analysed 5fC was indeed enriched over distal regulatory regions marked by H3K4me1. The screenshot in Fig. 3a taken from the genome browser shows an example of this striking association. However, in contrast to mESCs, 5fC in embryonic tissues is predominantly enriched over ‘active’ enhancers marked by H3K4me1 and H3K27ac (Fig. 3b and Additional file 1: Figure S7). We found this to be true in all tissues analysed, regardless of the presence or absence of TDG (although 5fC levels are higher in *Tdg* null embryos, relative peak enrichment is not). Notably, 5fC displays a relatively small enrichment over regions marked by tri-methylation of histone H3 lysine 4 (H3K4me3), a modification associated with promoter regions of active genes and previously reported to show a positive correlation with 5fC in mESCs (Fig. 3b) [12, 28].

We next asked whether 5fC enrichment was specific for regions marked by activating histone modifications. To this end, we analysed the distribution of 5fC peaks in the hindbrain at regions marked by tri-methylation of histone H3 lysine 36 (H3K36me3), which normally labels transcriptional elongation and hence gene bodies, or by tri-methylation of lysine 27 (H3K27me3), a repressive mark deposited by the PRC2 complex. Notably, 5fC shows very little enrichment over these regions (Fig. 3c). Taken together, these data show a strong and specific enrichment of 5fC at active enhancer regions during embryonic development. This constitutes a significant difference in the profile of 5fC between mESCs and embryonic tissues and could reflect a different need for dynamic regulation in developing embryos compared to the stem cell state.

### Tissue-specific cytosine formylation at developmental enhancers

**Fig. 4:**
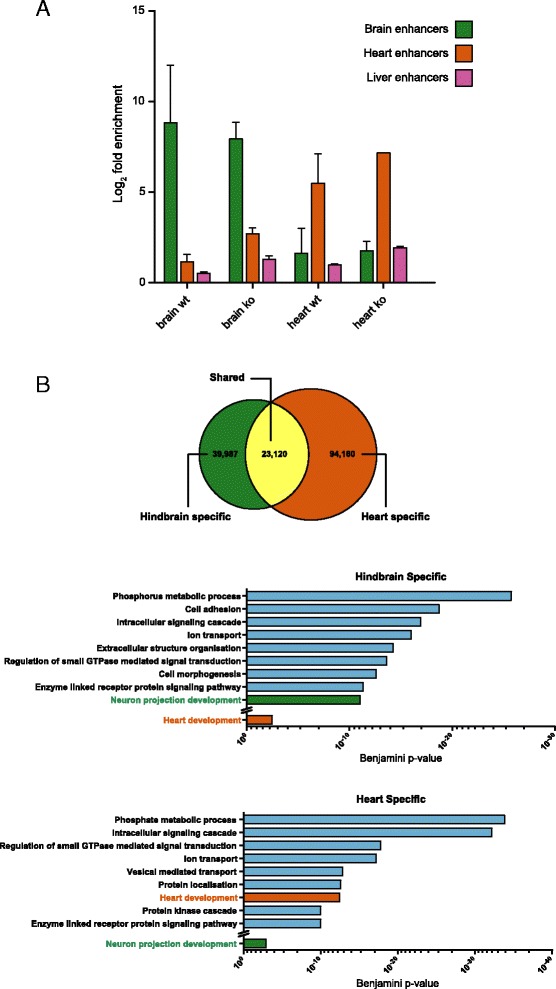
Tissue-specific 5fC formation at developmental enhancers. **a** Enrichment of 5fC peaks in the different tissues over tissue-specific enhancer regions. Log_2_ fold enrichment was calculated for each replicate individually using 10,000 randomisations in a simulation procedure implemented with the GAT. Plotted is the average enrichment with standard deviation. **b** Comparison of 5fC peaks detected specifically in the hindbrain and heart samples. Gene Ontology analysis was performed on genes in proximity (1 kb cutoff) of hindbrain-specific and heart-specific peaks

We next investigated whether the strong enrichment detected at active enhancers was regulated in a tissue-specific fashion. To answer this question, we analysed 5fC enrichment in heart-specific, hindbrain-specific and liver-specific enhancers, obtained from the ENCODE database (Additional file 2: Table S1), in the various tissues. We found that in hindbrain tissues from both WT and *Tdg* null embryos, 5fC was considerably enriched at brain-specific enhancers, compared to heart-specific and liver-specific ones. Similarly, heart-specific enhancers were specifically enriched in the heart samples. (Fig. 4a). These findings strongly indicate not only that there is a dynamic regulation of 5fC at the level of enhancers, but also that this complex mechanism is specifically focused at tissue-specific developmental enhancers. In line with this observation, TET-mediated active demethylation of developmental enhancers has been recently shown to be a conserved mechanism in vertebrate embryogenesis [29].

Finally, we investigated the relationship between tissue-specific 5fC formation and gene function. To this end, we subdivided 5fC peaks in the individual tissues from *Tdg* null embryos into shared and tissue-specific peaks. Notably, heart-specific 5fC peaks were enriched in the proximity of genes involved in heart development, whereas hindbrain-specific peaks were enriched proximal to genes involved in neuron development (Fig. 4b and example in Additional file 1: Figure S8). Interestingly we could not find a similar enrichment for liver-specific 5fC peaks, which may be due to the mixed endoderm/mesoderm lineage of this tissue [30]. Taken together, these results clearly indicate the presence of a tissue-specific role for 5fC formation at the level of genes involved in the development of the cognate tissues.

## Conclusions

We have generated the first in vivo genome-wide profile for 5fC in tissues obtained from WT and *Tdg* null E11.5 mouse embryos; namely from dissected heart, hindbrain and liver tissues and the carcass. While showing an overall similar profile to the ones reported in mESCs, with enrichment at enhancers and exons, we also find that 5fC is specifically enriched at active enhancers in the developing embryo. Moreover, we report clear tissue specificity behind the distribution of 5fC in developmentally related enhancers. This highlights a complex regulation of 5fC, balancing of TET oxidation and TDG excision, and thus suggesting a key role for dynamic demethylation at those sites. The relationship between this process and the striking phenotype displayed by *Tdg*-deficient embryos warrants future investigation.

## Methods

### Knockout mouse and tissue dissection

*Tdg* null mice were described in [21]. These mice contain a deletion of the coding sequence for the catalytic domain of TDG, which is replaced by a neomycin-resistant cassette. Genotyping was carried out using DNA isolated from the yolk sac as template with the following primers:

- TDG KO/WT forward: 5′-GGGCAGCAAGGATCTGTCTA-3′
- TDG KO/WT reverse: 5′-GCCAGTTCCTCTGACACTTAGC-3′

Mice were maintained as heterozygotes and intercrossed to generate homozygous knockout embryos for analysis. Tissues were collected from timed matings with the day of the copulation plug as E0.5. Embryos were dissected into hindbrain, heart, liver and carcass.

### DNA and RNA extraction

Embryonic tissues were lysed in RLT plus buffer (Qiagen), homogenised via centrifugation through a QIAshreddred column (Qiagen) at full speed for 2 min in a microcentrifuge. DNA and RNA were isolated using AllPrep DNA/RNA kit (Qiagen) following the manufacturer’s instructions.

### Mass spectrometry of DNA

A total of 100–300 ng of genomic DNA was treated with 5 U of DNA Degradase Plus (Zymo Research) for 3 h and analysed on a nanoHPLC combined with a Q Exactive mass spectrometer, as previously described [31].

### Pull-down method

Genomic DNA (500 ng in 10 mM Tris–HCl pH 8, 1 mM EDTA) was sonicated to an average of 250 bp with the Covaris M220 Focused-ultrasonicator. Synthetic spike-in controls were added after sonication to monitor the enrichment efficiency (full details on the spike-in oligos and conditions used are in Additional file 3). The fragmented DNA was incubated with a cleavable aldehyde reactive biotinylation probe [22] (400 μM), *p*-anisidine (100 mM) and NH_4_OAc (40 mM, adjusted to pH 5 with AcOH) in a final volume of 100 μL at 37 °C with a 65 °C heated lid for 24 h. The DNA was purified on silica-based spin columns (GeneJET PCR Purification Kit), with the inclusion of an additional wash step, before standard library preparation.

Streptavidin paramagnetic particles (50 μL, Promega Magnesphere PMPs) were washed with 1× binding buffer (5 mM Tris-HCl pH 7.2, 0.5 mM EDTA, 1 M NaCl, 0.05 % Tween-20) (four times 500 μL) and resuspended in 2× binding buffer (50 μL). The washed beads (50 μL), dIdC (2 μL of 1 μg/μL), spike-in control sequences (1 pg positive control; 10 pg negative control), DNA library and water were combined in a 1.5 mL low binding tube (Eppendorf DNA LoBind) to give 100 μL. The mixture was incubated at room temperature for 20 min, before the tube was placed on a magnetic rack and left for 5 min while the magnetic beads formed a pellet. Once the solution was clear the supernatant was discarded, and 400 μL 1× binding buffer was added to the tube. The beads were washed by rapidly rotating the tube 180° and allowing the beads to recollect against the magnet; this washing procedure was repeated four times. The beads were resuspended in 400 μL 1× binding buffer with vortexing and transferred to a new low binding tube before the washing procedure was repeated another four times. A final tube transfer and washing cycle was performed, giving a total of two tube changes and 12 washes. A final wash with Tris–HCl (10 mM) was performed, with the beads remaining on the magnet at all times due to poor pellet formation in this buffer.

The DNA was eluted from the beads by chemical cleavage of the probe with 100 μL of tris(2-carboxyethyl)phosphine (100 mM) and Tris–HCl (200 mM) shaken at 65 °C for 15 min. NaOAc (40 μL of 5 M, pH 5), ethanol (1 mL) and water (100 μL) were added to the isolated supernatant, and the mixture held at −80 °C for 45 min. The precipitated DNA was pelleted by centrifugation at 16,000 rcf for 45 min, washed with 70 % EtOH and centrifuged again at 16,000 rcf for 30 min. The pellet was air dried and dissolved in water (20 μL). At this point the enrichment efficiency was examined by qPCR of the control sequences spiked in before pull-down. One microliter of enriched DNA was diluted tenfold, from which 1 μL aliquots were amplified with primer sets for the positive and negative controls. Comparison to a non-enriched input gave the enrichment factor (Additional file 1: Figure S9 and Additional file 3). Although the enrichment was extremely high (~54,000-fold), there will be a proportion of false-positive regions arising from the excess of non-formylated DNA in genomic context.

### Library preparation and sequencing

Sequencing libraries were generated using the NEBNext library preparation modules. Fourteen cycles of PCR amplification were performed with the Illumina PCR Master Mix and PCR Primer Cocktail. Prior to sequencing, libraries were quantified by qPCR (KAPA Biosystems) and their fragment size profile analysed by TapeStation (Agilent). Sequencing was performed on an Illumina Nextseq 500, run in paired-end mode with 100 read cycles.

### Bioinformatic analysis

Raw sequencing files and peak files can be found in the GEO under accession number GSE77447. Data were adapter and quality trimmed using trim_galore v0.4.1 (bioinformatics.babraham.ac.uk/projects/trim_galore/) using default settings. Trimmed sequencing reads were aligned to mouse genome assembly NCBIM37 (mm9) using Bowtie2 v2.2.6 using default parameters [32] and was converted to BAM format using samtools v0.1.18 [33]. Peaks were called in the SeqMonk software (bioinformatics.babraham.ac.uk/projects/seqmonk/) using the MACS peak caller [34], with a *p* value cutoff of 1 × 10^−5^ and a fragment size of 200 bp. Data manipulations were facilitated by BEDTools [35] and data analysis was performed in Seqmonk or in R version 3.1.2 [36].

### Gene Ontology

Functional annotation enrichment analyses were performed using the Database for Annotation, Visualization and Integrated Discovery (DAVID) v6.7 [37].

## Abbreviations

5caC, 5-carboxylcytosine; 5fC, 5-formylcytosine; 5hmC, 5-hydroxymethylcytosine; 5mC, 5-methylcytosine; BER, base excision repair; BS-seq, bisulphite sequencing; CGI, CpG island; GAT, genome association tester; LC/MS, liquid chromatography/mass spectrometry; mESC, mouse embryonic stem cells; NPC, neural precursor cells; TDG, thymine DNA glycosylase; WT, wild type.

## Additional files

Additional file 1: Supplementary figures and supplementary figure legends. (PDF 841 kb) Additional file 2: Table S1. ENCODE datasets analysed. (PDF 53 kb) Additional file 3: Full details on spike-in control oligos. (PDF 226 kb)
