# Supplementary material for: In vivo genome-wide profiling reveals a tissue-specific role for 5-formylcytosine
Source: Genome Biol. 2016 Jun 29;17:141. doi: 10.1186/s13059-016-1001-5 (PMC4928330; doi:10.1186/s13059-016-1001-5)
Supplement: Additional file 1: — Supplementary figures and supplementary figure legends. (PDF 841 kb) [file 13059_2016_1001_MOESM1_ESM.pdf]

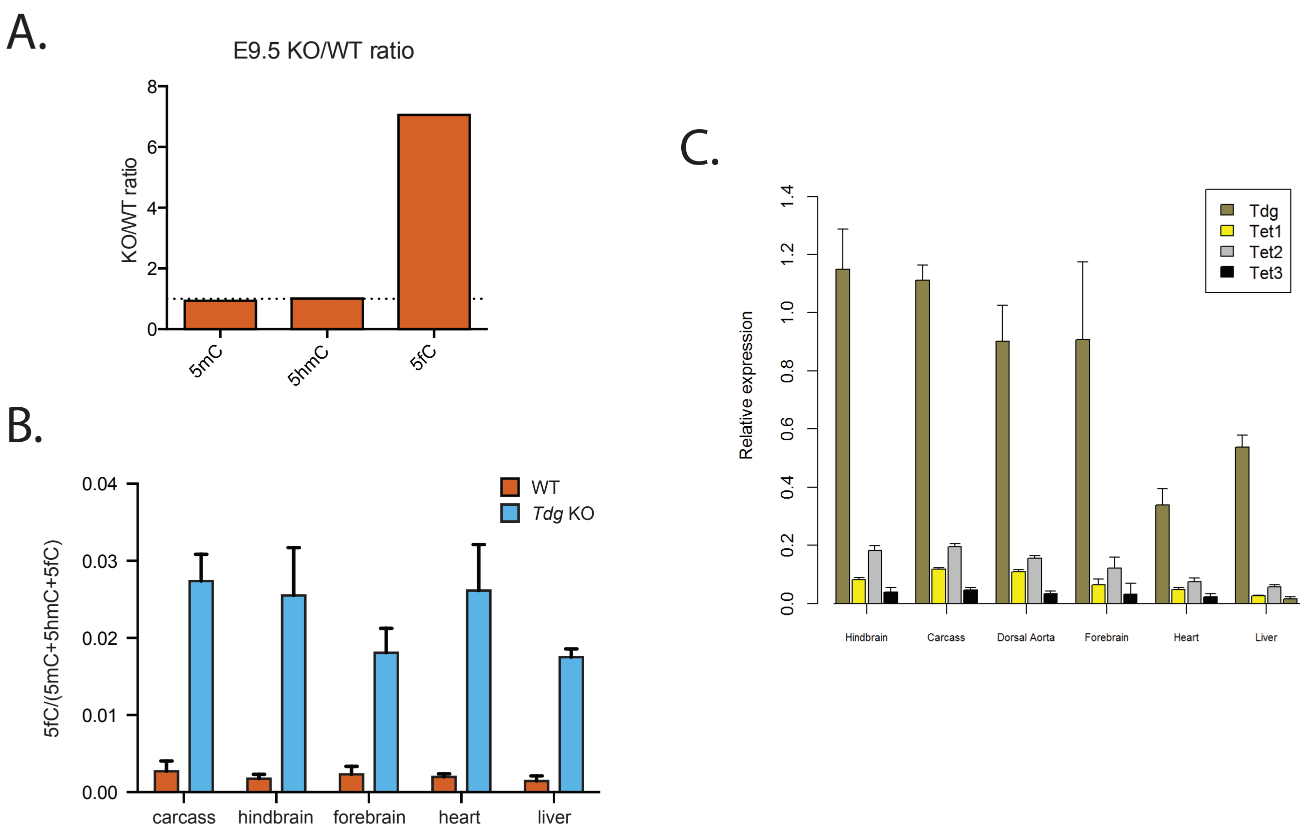

Figure S1. (A) Ratio of 5mC, 5hmC and 5fC levels between E9.5 WT and *Tdg* null embryos, as quantified by LC/MS. **(B)** LC/MS quantification of genomic 5fC levels in mid-gestation embryos. Displayed is average of at least three biological replicates with standard deviation. Results are expressed as ratio over all modified cytosines (5mC+5hmC+5fC). **(C)** mRNA levels for *Tdg* and *Tet* enzymes in tissues from E11.5 mouse embryos, as quantified by qPCR.

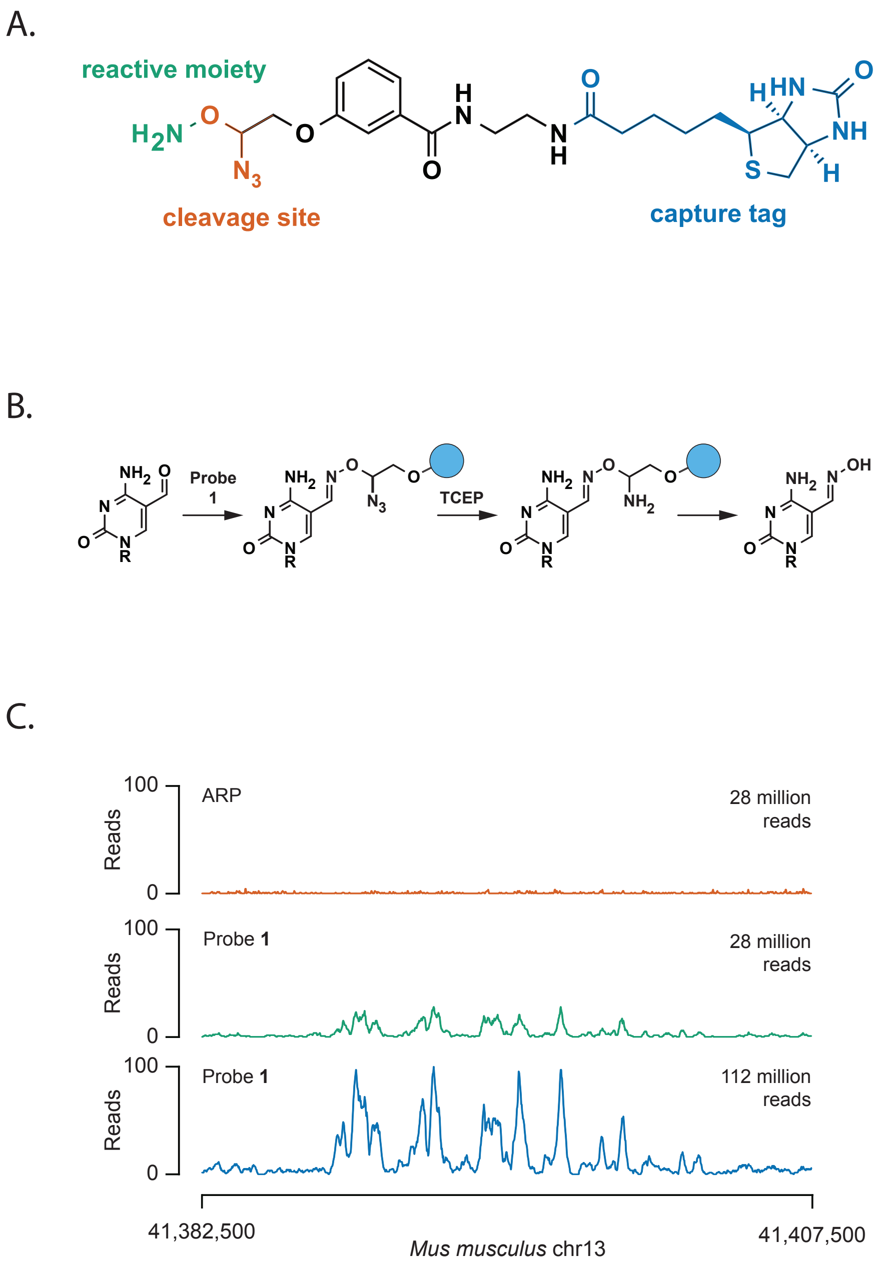

Figure S2. (A) The aldehyde reactive probe employed for genome-wide mapping of 5fC. Highlighted are the reactive oxyamine moiety, the azide-masked hemiaminal ether cleavage site and the biotin capture tag that enables enrichment with streptavidin-coated magnetic beads. **(B)** Scheme showing how the chemical probe may be cleaved by TCEP-mediated azide reduction. A Staudinger reduction of the azide unmasked a hemiaminal ether moiety, which spontaneously decomposes to yield 5-formyloximecytosine free from the biotin tag. **(C)** Genome browser screenshot showing improvements in signal to noise ratio using the new chemical probe for 5fC pull-down (Probe 1). Read counts are shown with a solid line. Signal to noise ratio is also improved by sequencing to a greater depth per sample.

A.

Tissue	wt or ko	Replicates	Litter pair
hindbrain	wt	4	a,b,c,d
heart	wt	2	e,f
liver	wt	2	e,f
carcass	wt	2	e,f
hindbrain	ko	4	a,b,c,d
heart	ko	2	e,f
liver	ko	2	e,f
carcass	ko	2	e,f

B.

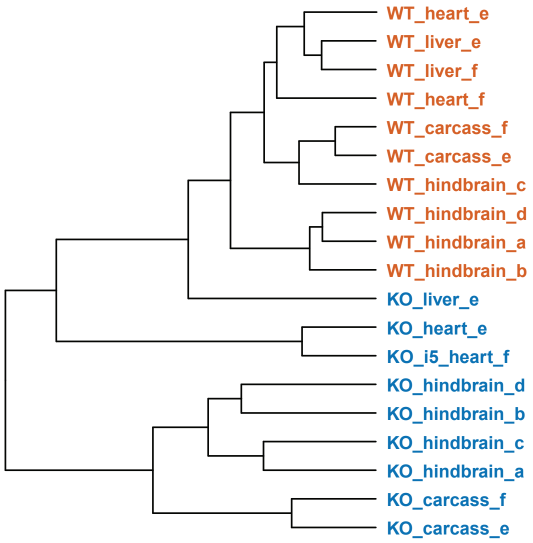

Figure S3. (A) Full list of tissues and biological replicates used and **(B)** unbiased hierarchical clustering of all samples.

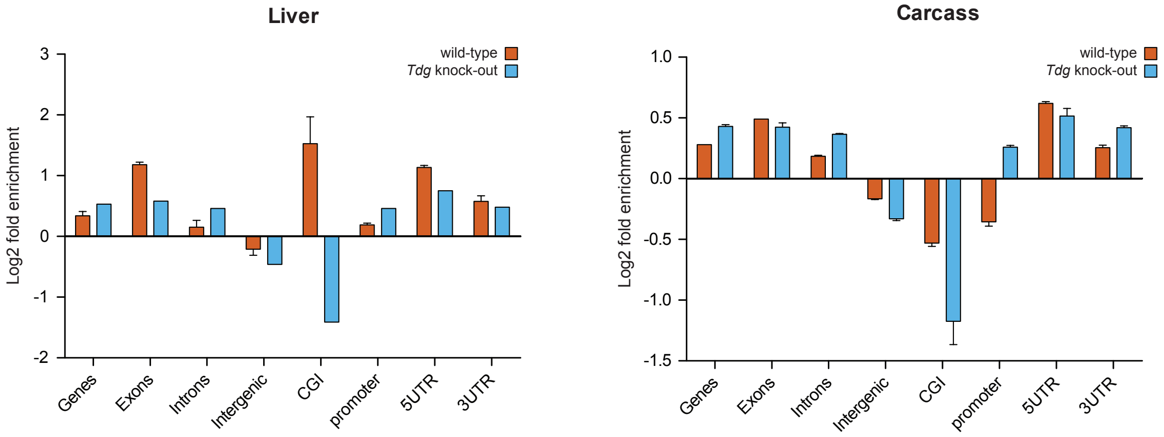

Figure S4. Enrichment of 5fC peaks over functional genomic features in E11.5 liver and carcass, respectively. Log_2 fold enrichment was calculated for each replicate individually using 10,000 randomizations in a simulation procedure implemented with the Genomic Association Tester (GAT).

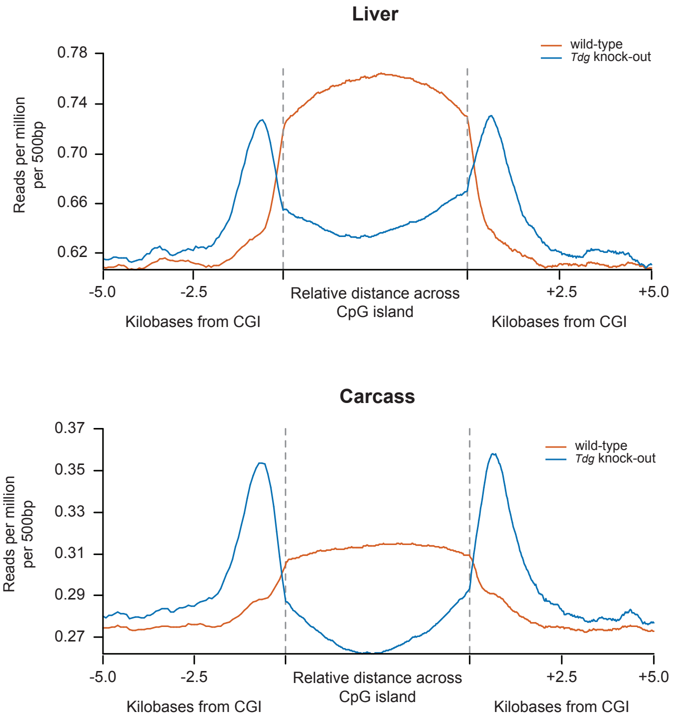

Figure S5. Trend plot showing 5fC profile over CpG islands (+/- 5kb) in the liver and carcass, respectively.

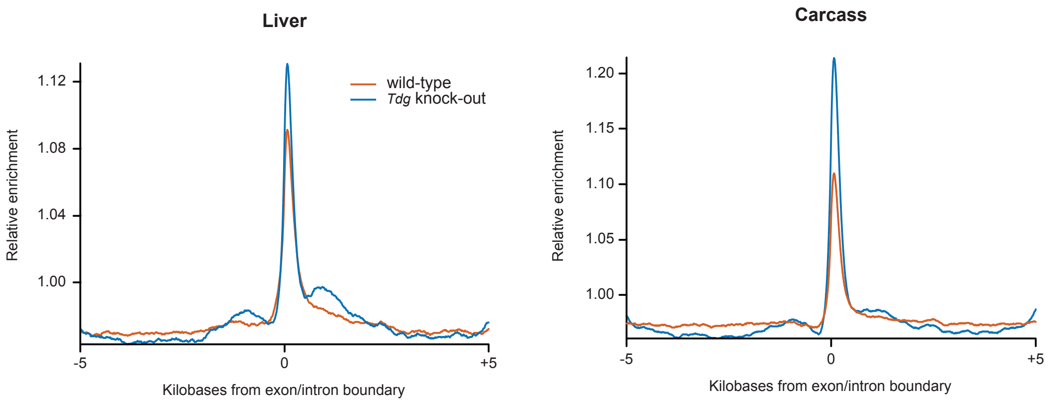

Figure S6. Trend plot showing 5fC profile over exon/intron boundaries (+/- 5kb) in the liver and carcass, respectively.

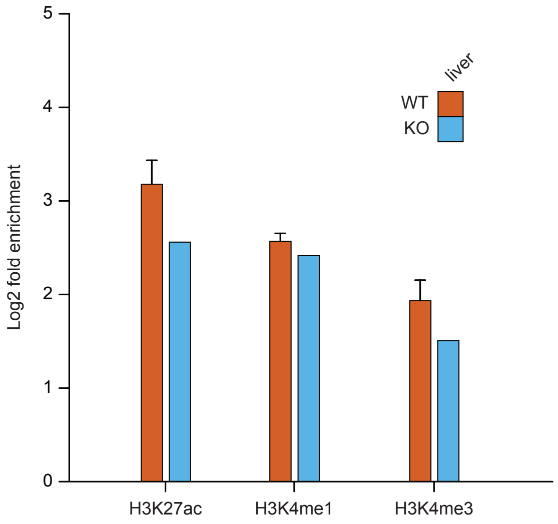

Figure S7. Fold enrichment of 5fC peaks in the liver over regions marked by H3K4me1, H3K4me3 and H3K27ac in the liver of E14.5 embryos. Log_2 fold enrichment was calculated for each replicate individually using 10,000 randomizations in a simulation procedure implemented with (GAT).

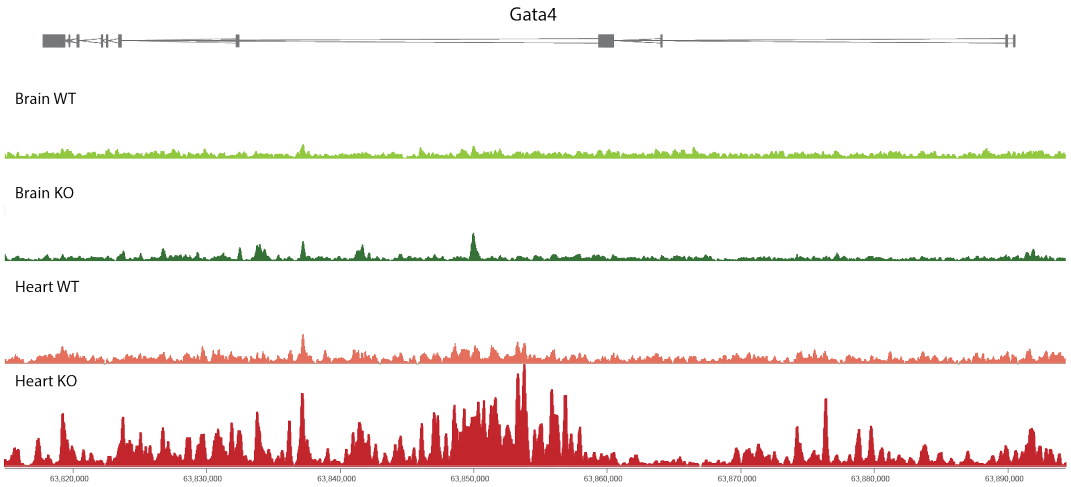

Figure S8. Screenshot showing signal for 5fC over the *Gata4* gene, a key factor in heart development.

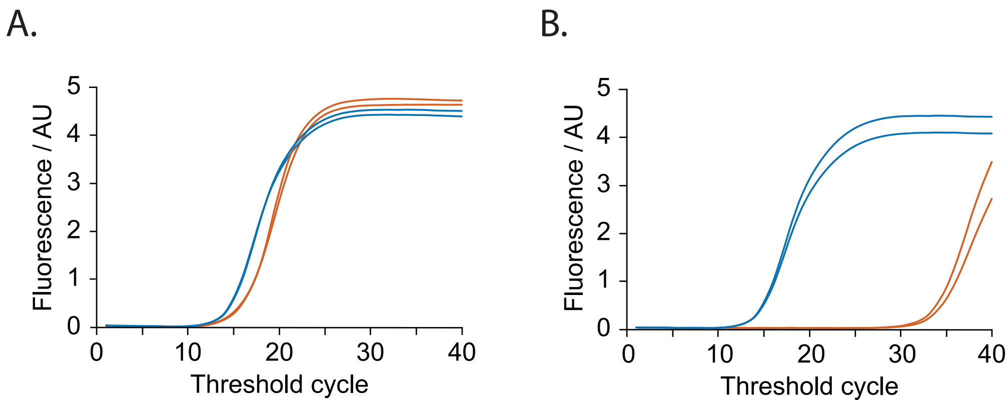

Figure S9. Representative enrichment quantification by qPCR. Amplification curves are colored blue for the positive control strand (containing 5fC) or red for the negative control strand. **(A)** Amplification curves for the non-enriched [input] spike-in control sequences **(B)** Amplification curves following the 5fC pull-down protocol.

Enrichment is calculated using the following equation:

$$\text{enrichment factor} = \frac{E^{C_{input\_positive} - C_{positive}}}{E^{C_{input\_negative} - C_{negative}}}$$

where E is the amplification efficiency of the control sequence, and C is the threshold cycle obtained from averaging technical replicates of the appropriate strand.
