# Supplementary material for: In vivo genome-wide profiling reveals a tissue-specific role for 5-formylcytosine
Source: Genome Biol. 2016 Jun 29;17:141. doi: 10.1186/s13059-016-1001-5 (PMC4928330; doi:10.1186/s13059-016-1001-5)
Supplement: Additional file 2: Table S1. — ENCODE datasets analysed. (PDF 53 kb) [file 13059_2016_1001_MOESM2_ESM.pdf]

Table S1. ENCODE datasets analysed

**Tissue**	**Antibody employed**	**UCSC Accession**
Whole brain	H3K27ac	wgEncodeEM002491
Whole brain	H3K4me1	wgEncodeEM002492
Whole brain	H3K4me3	wgEncodeEM002493
Whole brain	H3K27me3	wgEncodeEM002725
Whole brain	H3K36me3	wgEncodeEM002718
Heart	H3K27ac	wgEncodeEM002503
Heart	H3K4me1	wgEncodeEM002504
Heart	H3K4me3	wgEncodeEM002505
Liver	H3K27ac	wgEncodeEM002571
Liver	H3K4me1	wgEncodeEM002573
Liver	H3K4me3	wgEncodeEM002572
