# Supplementary material for: In vivo genome-wide profiling reveals a tissue-specific role for 5-formylcytosine
Source: Genome Biol. 2016 Jun 29;17:141. doi: 10.1186/s13059-016-1001-5 (PMC4928330; doi:10.1186/s13059-016-1001-5)
Supplement: Additional file 3: — Full details on spike-in control oligos. (PDF 226 kb) [file 13059_2016_1001_MOESM3_ESM.pdf]

Spike-in controls

Q36: 5’ to 3’; 100 bases; single 5fC incorporated during PCR with d5fCTP
CTCACCCACAACCACAAACAATTTAAATAATATTAAATAATATTAATATA
TTAT**CG**ATTAAATAATAATTAATTAATATTGGTTGGATGGTAGATGGTGA

Q78: 5’ to 3’; 100 bases; single C incorporated during PCR with canonical dNTP mix
ATCCACATCATTACCCATCCATTTAAATAATATTAAATAATATTAATATAT
TAT**CG**ATTAAATAATAATTAATTAATATTTGATGTGATGGGTGGTATGG

The sequences for which synthetic primers are used for amplification are underlined (and therefore the Cs in the primer region do not become 5fC in Q36), sites of 5fC incorporation (Q36) or C incorporation (Q78) are in bold.

Two spike-in sequences were used to assay the efficiency of the 5fC pull-down. The Q36 sequence was a positive control, containing a single 5fC per strand, while the Q78 sequence served as a negative control and contained only canonical DNA bases.

The amplification efficiency of the two strands was checked by running dilution series over six-orders of magnitude, and was found to be 89.1% and 88.2% for Q36 and Q78 respectively, with the linear amplification range extending from 10 pg to 0.0001 pg. The controls were spiked-in at 1 pg (Q36) or 10 pg (Q78). The equation used to determine the enrichment efficiency from qPCR data (Additional File 1: Figure S9) is described as follows:

$$\text{enrichment factor} = \frac{E^{C_{input\_positive} - C_{positive}}}{E^{C_{input\_negative} - C_{negative}}}$$

where *E* is the amplification efficiency of the control sequence, and *C* is the threshold cycle obtained from averaging technical replicates of the appropriate strand.
